# 

**Published:** 1845-12

**Authors:** 


### CONTENTS

OF THE

### MEDICAL EXAMINER.

NEW SERIES—NO. XII.—DECEMBER, 1845.

### ORIGINAL COMMUNICATIONS.

Parisian Medical Schools. By Alexander Wilcocks, M.D. - - - 713
Extract of a letter from William B. Diver to Professor Dunglison, - - 714
A case of Typhoid Fever, in which the Hydriodate of Potassa was successfully administered. By C. B. Voigt, M. D, - - - 715

### BIBLIOGRAPHICAL NOTICES.

Essays on Patholgy and Therapeutics, being the substance of a Course of Lectures delivered by Samuel Henry Dickson, M. D., Professor of the Institutes and Practice of Medicine in the Medical College of the State of South Carolina, - - - - - - 716
Urinary Deposites, their Diagnosis, Pathology, and Therapeutical indications. By Golding Bird, A. M., M. D., Assistant Physician to, and Lecturer on Materia Medica at, Guy's Hospital, etc. etc. etc. 723

A Manual of Auscultation and Percussion. By M. Barth, Agrégé to the Faculty of Medicine of Paris, &c. &c., and M. Henry Roger, Physician to the Bureau Central of the Parisian Hospitals, &c. &c. Translated, with additions, by Francis G. Smith, M. D., Lecturer on Physiology in the Philadelphia Medical Association, &c. &c. 730

A Practical Treatise on Diseases of the Sexual organs ; adapted to popular and professional reading, and the exposition of Quackery, professional and otherwise. By Edward H. Dixon, M. D., etc. etc. 730

The Anatomical Remembrancer ; or Complete Anatomist; containing a concise description of the Bones, Ligaments, Muscles, aud Viscera, &c. &c. From the Second London edition, revised, - - 731

### EDITORIAL.

The New York Medical and Surgical Reporter. Edited by Clarkson T. Collins, M. D., etc, - - - - - - - - - 732

Medical Classes in Philadelphia, - - - - - - - - 733

Clinical Instruction, - - - - - - - - - 733

### RECORD OF MEDICAL SCIENCE.

Report on the Progress of Surgery. By Henry Ancell, Esq, M. R. C. S. E.

Nature and Treatment of Tumours, - - - - - - 736

Treatment of cancerous tumours of the breast without operation, - 739

Temperature in operations, and the treatment of surgical diseases, 739

Diagnosis and treatment of syphilitic diseases, - - - - - 741

Thoracic surgery, - - - - - - - - - - 741

Case of injury of the chest, with hernia of the right lung, - - - 742

Abdominal surgery, - - - - - - - - - - - - 742

Treatment of fistula in ano by ligature, - - - - - - - 744

Cure of aneurism by pressure, - - - - - - - - 744

Tracheotomy, - - - - - - - - - - - 747

Operation for abscess in the jaw, - - - - - - - - 748

Two cases of puncture of the intestines to relieve the agony of distension in supposed internal strangulation, - - - - - - 749
A few Observations on the use of large doses of Quinine in the treatment of Bilious Remittent Fever. By W. J. Tuck, M. D., of Memphis, Tenn, - - - - - - - - ' - - 749
Passage of Metallic Mercury into the blood and various organs of the body, - - - - - - - - - - - 756
Improved Life Preserver, - - - - - - - - - - 756
Great Malformation of the Heart; a single Auricle and Ventricle, . - 757
Case of "Glanders" in the Human Subject.—(Equinia.) By C. Smallwood, M. D. St. Martin, - - - - - - - - 758
On Goitre in Switzerland, - - - - - - - - - 760
On a Venous Pulse in the Veins on the back of the hand. By M. Martin-Solon, - - - - - - . - - - - 761
Phenomena of Respiration. By M. Vierordt, - - - - - 762
Pulmonary Fistula, consecutive on a scrofulous necrosis. By M. Grapin, 763

